# Arterial Remodeling in B-Type Natriuretic Peptide Knock-Out Females

**DOI:** 10.1038/srep25623

**Published:** 2016-05-10

**Authors:** Sara J. Holditch, Claire A. Schreiber, John C. Burnett, Yasuhiro Ikeda

**Affiliations:** 1 Department of Molecular Medicine, Mayo Clinic, College of Medicine, Rochester, MN, USA; 2 Cardiorenal Research Laboratory, Division of Cardiovascular Diseases, Departments of Medicine and Physiology, Mayo Clinic, College of Medicine, Rochester, MN, USA

## Abstract

Sexual dimorphisms are recognized in cardiovascular conditions such as hypertension, stroke, thrombosis and vasculitis. B-type natriuretic peptide (BNP) is a guanylyl cyclase A (GC-A) agonist. The anti-hypertensive, vasodilatory, anti-fibrotic, and anti-hypertrophic properties of BNP are well established in male animal models. Although circulating BNP levels are higher in women, when compared to age-matched men, the cardiovascular protective propensity of BNP in females is poorly understood. We assessed the cardiovascular consequences of BNP deletion in genetically null (Nppb−/−) female rat lines. Throughout the study, blood pressure (BP) remained uninfluenced by genotype, and cardiorenal consequences of BNP knock out remained minor. Unexpectedly, approximately 60% of Nppb−/− females developed mesenteric polyarteritis-nodosa (PAN)-like vasculitis in their life span, some as early as 4 months of age. Mesenteric lesions involved intense arterial remodeling, progressive inflammation, occluded lumens, and less frequently intestinal necrosis and multiple visceral arterial aneurysms. Cumulative pathologies resulted in a significant decline in survival of the Nppb−/− female. This study highlights BNP’s vasoprotective propensity, bringing to light a possible sex specific difference in the cardiovascular protection provided by BNP. Defects in the BNP/GC-A/cGMP pathway may play a role in arteriopathies in women, while GC-A agonists may provide effective therapy for arteritis.

Gender differences, and thus sex hormones, are recognized factors involved in the frequency, clinical manifestation, and mortality of cardiovascular disease^1^. For example, endogenous estrogens maintain vasodilation, contributing to pre-menopause blood pressure control^2^, whereas an earlier age of menopause is linked to earlier onset and increased growth rate of aortic aneurysms^3^, among other cardiovascular morbidities. On the other hand, estrogen replacement therapies (ERT) can result in thrombosis^4^ and may increase the risk of heart disease and stroke^5^. A better understanding of how sex influences the molecular mechanisms involved in the etiology of cardiovascular disease may identify factors for targeted pharmacological intervention.

B-type natriuretic peptide (BNP) is a cardiac hormone in the natriuretic peptide family, integral in protecting endothelial function^6^ and maintaining pressure and volume of the cardiovascular system^7^. Once in circulation BNP binds principally natriuretic peptide receptor A (Npr-A)^8^, a particulate guanylyl cyclase GC-A, leading to an increase of the intracellular secondary messenger, cyclic-GMP (cGMP)^9^. In circulation, BNP can also bind natriuretic peptide receptor C (Npr-C), nicknamed the clearance receptor, which does not lead to an increase in cGMP^10^. The BNP/GC-A system is also implicated in neovascularization of ischemic tissues^11^,^12^,^13^. Likewise; a recent study demonstrates the importance of the GC-A pathway in embryonic vascular development^14^.

Others have shown male mice genetically null for BNP develop multi-focal cardiac fibrosis^15^. Inversely, male BNP-transgenic mice have significantly reduced blood pressure and protection against a variety of cardiorenal assaults^16^,^17^,^18^. Recently published, BNP deletion in male rats leads to adult-onset hypertension with myocardial hypertrophy, cardiac fibrosis and progressive hypertensive nephropathy^19^. Moreover, sustained BNP over-expression by cardiotropic adeno-associated vectors prolongs survival of male spontaneously hypertensive rats, and prevents the development of hypertensive heart disease^20^,^21^. Clinically, circulating BNP concentrations are significantly elevated in women compared to age-matched men, in both healthy^22^ and pathophysiological^23^ states. Additionally, there is an increase in BNP levels in both men and women with age^24^ corresponding to the increasing prevalence of cardiac disease in mature patients^24^. However, there is a lack of studies addressing this gender difference, or the protective role of BNP specific to women.

Here, our goal was to address this gap by characterizing the cardiovascular consequences of BNP deletion in a female rat. To elucidate the functional significance of BNP knockout, we characterized two genetic BNP-null (Nppb−/−) female rat lines^19^,^25^ against BNP-intact age matched control female rats. Our study highlights a novel and previously unrealized role for BNP in the etiology of, and possibly the sexual dimorphic nature of vascular diseases.

## Results

**Figure 1:**
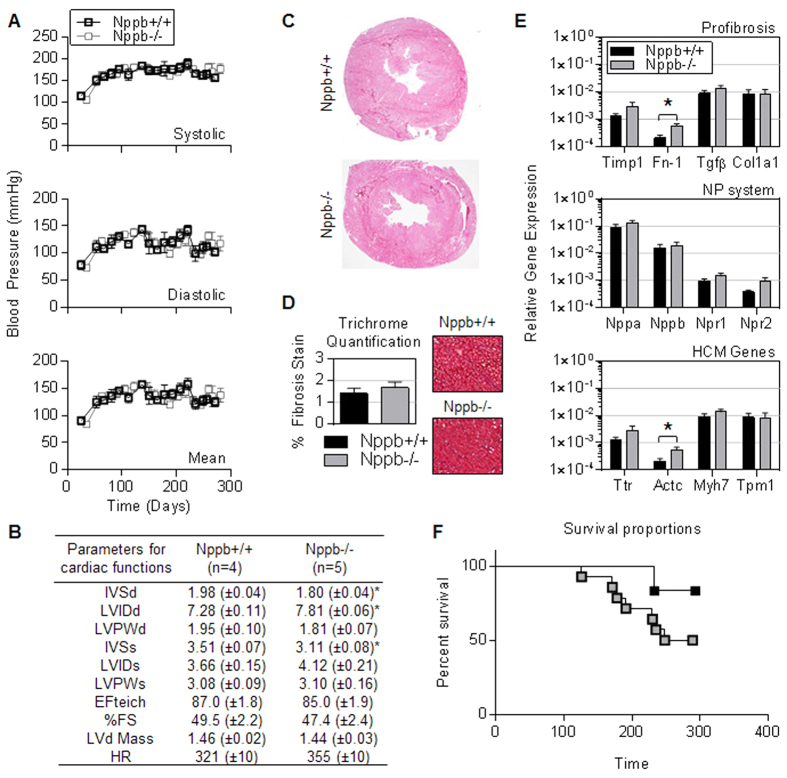
Preserved cardiac function in aged BNP knockout females. (**A**) Noninvasive systolic, diastolic, and mean blood pressure measurements in Nppb−/− (n = 14) and age-matched control Nppb+/+ (n = 6); measurements were initiated at 25 days of age and monitored bimonthly through study termination at 9 months. Genotypes were assayed at the same time-point, however due to similarity of the data sets, BP lines are graphed staggered for visual ease. (**B**) Echocardiographic assessment of cardiac remodeling of surviving genotypes were measured at nine months, Nppb−/− (n = 5), Nppb+/+ (n = 4). (**C**) Representative myocardial histology of Nppb−/− and age-matched Nppb+/+ (**D**) and quantification of Massons Trichrome Staining at nine months (n = 5, n =  4) Nppb−/− and Nppb+/+ respectively, with cardiac sections at 40× magnification. (**E**) RT-PCR quantification at three months of Profibrosis associated genes; Collagen type 1a1 (Col1a1), Fibronectin-1 (Fn1), Transforming growth factor-β (TGFβ), Tissue inhibitor metalloprotease-1 (Timp1), Natriuretic Peptide (NP) system associated genes; Atrial Natriuretic Peptide (Nppa), B-type Natriuretic Peptide (Nppb), Natriuretic Peptide Receptors 1 and 2 (Npr1, Npr2, respectively) and Hypertrophic Cardiomyopathy (HCM) associated genes; Transthyretin (Ttr), Alpha Cardiac Actin (Actc), Myosin heavy chain 7 (Myh7) and Tropomyosin 1 (Tpm1) transcripts, housekeeping gene Beta Actin corrected. Nppb−/− (n = 6) and age-matched control Nppb+/+ (n = 6). *P < 0.05 vs Nppb+/+ by *t* test, data represent the mean ± SEM. (**F**) Survival curve of Nppb−/− (n = 14) and age-matched control Nppb+/+ (n = 6) over nine months; survival curve compared by log-rank (Mantel-Cox) test.

Preserved cardiac function in BNP knockout females. Every two weeks from 21 through 270 days of age BNP knockout females (Nppb−/−) and age-matched controls (Nppb+/+) were monitored for systolic, diastolic, and mean blood pressure (BP). No notable difference was seen in BP measurements (Fig. 1A). Echocardiographic analysis of cardiac output found no significant difference between genotype at 9 months, although a marginal cardiac dilation was suggested by increased left ventricular internal dimension and reduced septal wall thickness (Fig. 1B). Additionally, histology at nine months suggested similar myocardial pathology between genotypes (Fig. 1C). Although increased cardiac fibrosis has been observed in both male BNP knockout mice and rats^15^,^19^, Trichrome staining of the female knock out cardiac tissues at 9 months identified no significant increase in cardiac fibrosis (Fig. 1D). Moreover, BNP knock out resulted in no notable changes in the transcript levels of cardiac specific profibrotic genes or hypertrophic myocardial signaling pathway genes, aside from marginal increases in fibronectin-1 (Fn-1) and alpha cardiac actin (Actc) expression in Nppb−/− (Fig. 1E). Nevertheless, a decline in Nppb−/− survival was noted by 8 months (Fig. 1F).

**Figure 2:**
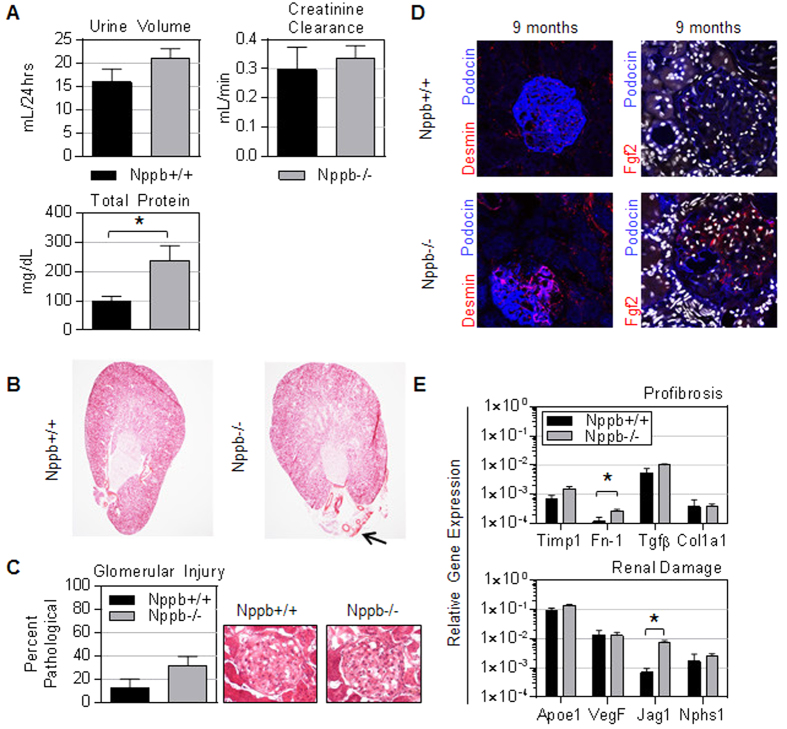
BNP deletion does not lead to marked renal pathology. (**A**) Urine volume, calculated creatinine clearance, and urinary excreted protein at nine months were measured in Nppb−/− (n = 5) and age-matched control Nppb+/+ (n = 5). (**B**) Representative hematoxylin and eosin stained renal sections of Nppb−/− and Nppb+/+ (Black arrow indicates noticeably enlarged artery present in Nppb−/− section), and (**C**) quantification of renal pathology in Nppb−/− (n = 5) and Nppb+/+ (n = 4). (**D**) (Left) Representative microphotographs of immunofluorescent renal sections from 9 month Nppb−/− and 9 month Nppb+/+; anti-Desmin (Desmin, Red), and anti-Podocin (Blue). (Right) Representative Nppb−/− and Nppb+/+ glomeruli immunofluorescent imaging of anti-basic fibroblast growth factor 2 (Fgf2, Red) and anti-podocin (Blue), and nuclear counter stain, DAPI (White). (**E**) RT-PCR quantification of Collagen type 1a1 (Col1a1), Fibronectin-1 (Fn1), Transforming growth factor-β (TGFβ), Tissue inhibitor metalloprotease-1 (Timp1), Atrial Natriuretic Peptide (Nppa), B-type Natriuretic Peptide (Nppb), Natriuretic Peptide Receptors 1 and 2 (Npr1, Npr2, respectively) and Renal damage associated genes; Apolipoprotein E (Apoe1), Vascular endothelial growth factor (VegF), Jagged1 (Jag1) and Nephrosis 1 (Nphs1) transcripts are corrected to house keeping gene GAPDH. Nppb−/− (n = 6) and age-matched control Nppb+/+ (n = 6). *P < 0.05 vs Nppb+/+ by *t* test.

Genetic BNP ablation leads to modest glomerular injury in females. Interrogating the renal consequence of BNP deletion revealed a mild renal phenotype in aged Nppb−/−. At 9 months, Nppb−/− had significantly elevated urinary total protein (Fig. 2A). However, Nppb−/− experienced relatively uninfluenced creatinine clearance, suggesting sufficient renal function at advanced ages (Fig. 2A). HE staining of renal sections revealed no marked difference in renal architecture between genotypes (Fig. 2B), although a trend of increased glomerular injury was observed at 9 months in Nppb−/−(Fig. 2C). Qualitatively, immunohistochemistry suggested more prominent glomerular remodeling in Nppb−/− tissues, compared to age-matched Nppb+/+ renal sections, with decreased podocin signals in podocytes, increased Desmin-positive mesangial cells, and basic-fibroblast growth factor 2-positive fibroblasts (Fig. 2D). Gene expression confirmed relatively minor increases in profibrotic pathways and injury markers in Nppb−/− kidneys (Fig. 2E).

**Figure 3:**
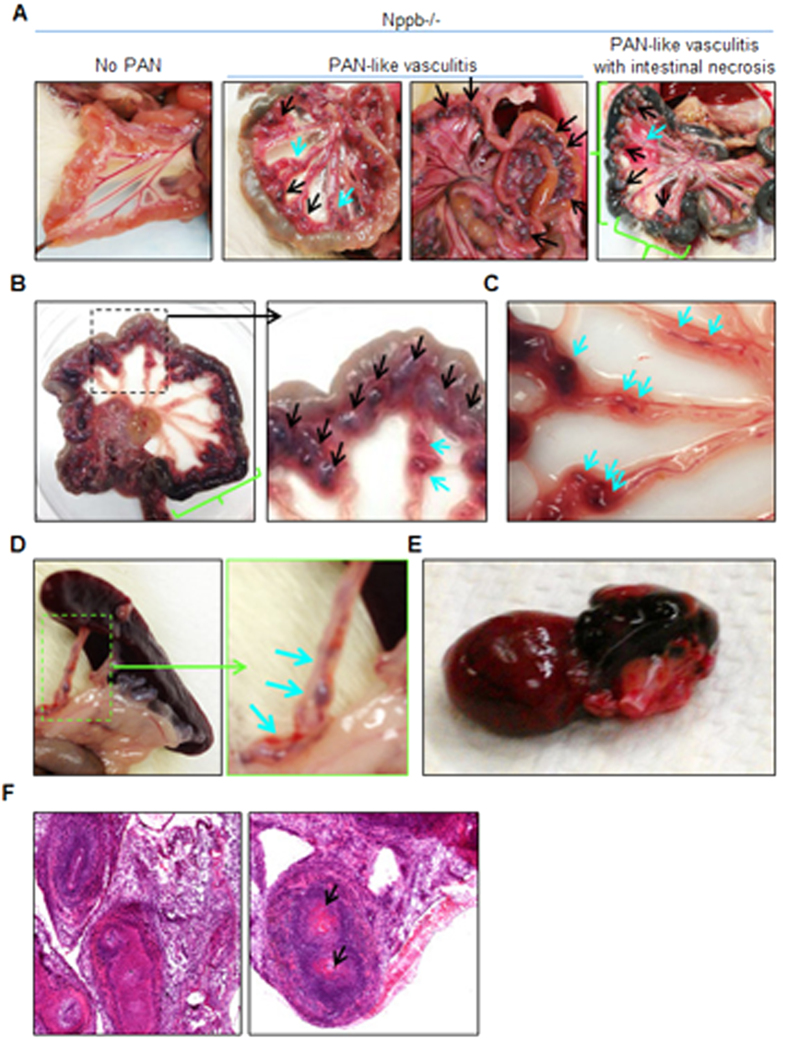
Polyarteritis like lesions, and arteriopathies in Nppb−/− females. (**A**–**C**) Autopsy of moribund Nppb−/− females revealed mesenteric arterial nodes (black arrows) with partial intestinal necrosis (green bracket) and mesenteric arteries with multiple aneurysms (blue arrows). (**D**) Aneurysms found in splenic arteries and (**E**) epicardial hemorrhage in Nppb−/−. (**F**) HE staining demonstrated marked arterial wall thickening, inflammation of the affected arterial wall, and artery occlusion by possible fibrin plug or thrombi (black arrows).

Decreased survival associated with multiple mesenteric vasculitis and necrotic gut. As Nppb−/− survival declined, autopsy of moribund animals revealed a significant incidence of mesenteric vasculitis with enlarged and tortuous mesenteric arteries (Fig. 3A,B, indicated by black arrows), similar to polyarteritis nodosa (PAN); a phenotype not observed in Nppb−/− males^19^. Between ages 4 and 9 months, Nppb−/− females developed PAN-like mesenteric vasculitis, commonly with intestinal necrosis or colitis (Fig. 3A, right panel, Fig. 3B, indicated by green lines) and multiple mesenteric aneurysms (Fig. 3A–C, indicated by blue arrows). A smaller subset of Nppb−/− females developed splenic arterial aneurysms (Fig. 3D, inset), and fewer yet developed epicardial hemorrhages (Fig. 3E) or stroke-like, sudden onset, neurological symptoms. Histology of PAN-like mesenteric lesions revealed intense arterial wall thickening, inflammation in the affected arterial wall and surrounding tissues (Fig. 3F), and some arteries nearly occluded by fibrin plugs and or thrombi (Fig. 3F, black arrows).

**Figure 4:**
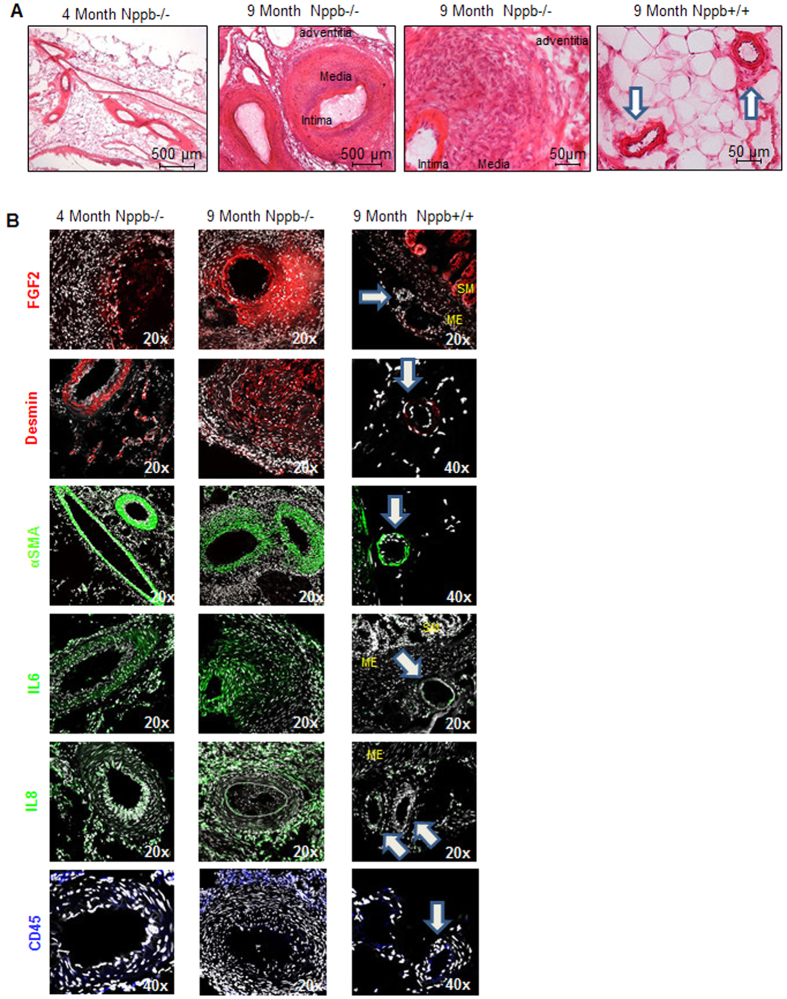
Histology of the mesenteric arteries in Nppb−/− at 4 and 9 months. (**A**) H&E analysis of progressive mesenteric arterial lesions in Nppb−/− . Mesenteric tissues obtained from animals of early and late symptom onset; 4 and 9 months in Nppb−/− vs 9 month Nppb+/+ (Control arteries highlighted by white arrows). (**B**) Factors involved in Nppb−/− mesenteric arterial remodeling: representative immunofluorescent microphotographs from 9 month Nppb−/− (n = 4), 4 month Nppb−/− (n = 3), and 9 month Nppb+/+(n = 3) sections, out of an average of 5 images per animal/section of mesenteric sections are shown. Specific antibodies include anti-Fgf2 (red), anti-Desmin (red), anti-α-SMA (green), anti-IL6 (green), anti-IL8 (green), and anti-CD45 (blue). Nuclei were counterstained by DAPI (white). SM; smooth muscle, ME: Mesentery, Control arteries highlighted by white arrows.

We then analyzed the progression of PAN-like lesions using the earliest and latest symptomatic time points; 4 month and 9 month aged Nppb−/− and 9 month control Nppb+/+ tissues. Histological analysis identified striking differences. Mesenteric arterial wall thickening was apparent at 4 months in Nppb−/−, however without notable inflammation (Fig. 4A, Left). By 9 months mesenteric arteries of Nppb−/− had intensive remodeling, characterized by thickening of the arterial wall, primarily the tunica media, and to a lesser degree in the tunica intima (Fig. 4A, middle panels). Arterial wall thickening was identified as a result of myofibroblast-like smooth muscle cell hyperplasia, and medial hypertrophy involving enlarged myocytes with distended nuclei and immune infiltration (Fig. 4A middle panels). No notable arterial remodeling, or increased arterial wall mass were present in 9 month old Nppb+/+ controls (Fig. 4A, right panel).

Immunohistochemistry of cellular populations present in PAN-like lesions between ages and genotypes identified increased anti-Fgf2 and anti-Desmin staining, identifying fibroblasts and smooth muscle cell populations, respectively, within and surrounding nodes, absent from control tissues (Fig. 4B). Significantly increased smooth muscle mass was evident by α-SMA staining, again absent from control Nppb+/+ mesenteric arteries (Fig. 4B). Prominent anti-IL6 and anti-IL8 staining, suggestive of wide-spread inflammation, was present in 9 month-old Nppb−/− PAN-like lesions. Enhanced inflammation was also supported by increased anti-CD45-positive T cell infiltration in the tunica intima of Nppb−/− nodes with inflammation not present in Nppb+/+ tissues (Fig. 4B).

Systemic BNP over-expression reduces the incidence of **PAN-like lesions** in female BNP knockout rats. To verify the direct role of BNP in the development of PAN-like arteritis in BNP KO rats, we transduced BNP KO female pups with adeno associated viral vectors (AAV) expressing rat proBNP^19^,^20^, and assessed the gross incidence of PAN-like lesions. Though cellular analysis was not undertaken to identify cell populations influenced by BNP supplement, we found reduced incidence of PAN-like lesions in BNP-treated female rats (17%, compared to 60% of untreated BNP knockout female littermates).

**Figure 5:**
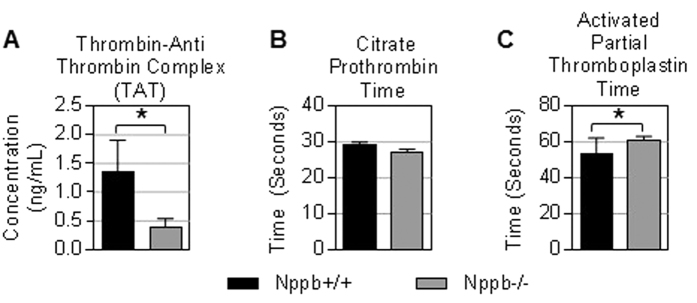
Coagulation component in aged Nppb−/− PAN. (**A**–**C**) Serum analysis of Thrombin-Anti Thrombin Complex, Activated Partial Thromboplastin Time, and Citrate Prothrombin Time are shown. Nppb−/− (n = 5) and age-matched control Nppb+/+ (n = 5). *P < 0.05 vs Nppb+/+ by *t* test.

Coagulation dysregulation in Nppb−/− female rats. Nppb−/− histopathology identified a subset of PAN-like lesions occluded with fibrin-like plugs (Fig. 3F). Additionally, moribund Nppb−/− females displayed other pathologies suggestive of enhanced coagulation processes, such as epicardial hemorrhage and sudden onset stroke-like neurological symptoms. From these observations, we speculated that genetic removal of BNP may have influenced an abnormal coagulation environment in females. In contrast to our expectations, the circulating concentration of thrombin-antithrombin complex (TAT) was found to be significantly lower in Nppb−/− rats, suggesting reduced activation of the coagulation pathway (Fig. 5A). Additionally, activated partial thromboplastin time (APTT) was significantly prolonged in Nppb−/− females (Fig. 5C), while citrate prothrombin time was found to be uninfluenced by genotype (Fig. 5B). These data suggest that genetic BNP ablation may influence the intrinsic and common coagulation pathways in females.

## Discussion

Androcentric reports of mouse models show that BNP knockout results in cardiac fibrosis^15^. We recently reported that male BNP knockout rats develop progressive, adult-onset hypertension, cardiac hypertrophy, and hypertensive nephropathy^19^ without vasculitis. Here, in the BNP knock out female littermates, BNP appears to play a less vital role in the etiology of hypertension, and cardiorenal remodeling. However, BNP knockout females do experience decreased survival, as a result of cumulative pathologies; early onset mesenteric vasculitis, visceral arterial aneurysms, and necrotic gut. Thus, our data set suggests that BNP may provide a specific cardiovascular protective propensity, unique to each sex.

Sex differences affect the control of blood pressure. Typically, men have higher blood pressure than age-matched premenopausal women^26^, although the prevalence of hypertension is greater in older women (60 years and older) than age-matched men^27^. Our previous study^19^ along with our current data set, reveal a sex-specific difference in the consequence of BNP deletion on blood pressure control. In females, the removal of BNP resulted in no notable effect on blood pressure. This observation is consistent with a mouse model defective in both eNOS and COX1, which demonstrates blood pressure elevations in males, but not in females^28^. Intriguingly, ovariectomy results in increased blood pressure in Dahl salt-sensitive (Dss) rats, supporting the blood pressure-lowering effects of female hormones^29^. Endothelial produced C-type natriuretic peptide (CNP) binds natriuretic peptide receptor, Npr-B/GC-B, in addition to sharing the same clearance receptor as BNP, Npr-C, a GC-independent receptor. CNP is also implicated in the maintenance of normal blood pressure in females^30^. It is possible that the effect of BNP deletion on blood pressure maintenance is compensated for by female hormones or CNP in female BNP knockouts.

Spontaneous polyarteritis nodosa (PAN), a similar presenting mesenteric arteriopathy, occurs in approximately 10% of normal laboratory rats late in life, with the average age being 28 months old^31^. Intriguingly, sustained estrogen treatment accelerates PAN onset in female rats to 12–18 month of age^32^. Experimental renal hypertension with high salt diet rapidly induces PAN in mesenteric, coronary, peripheral and/or pancreatic arteries in female rats by 4 months (30% of hypertensive and 0% of normotensive rats)^33^,^34^. Similarly, high salt diet in female Dss, but not Dahl salt-resistant rats, induces hypertension and PAN-like lesions by 4 weeks^35^. On the other hand, male spontaneously hypertensive rats (SHRs) with hypertension show an 82% incidence of PAN, whereas only 18% of age-matched hypertensive female SHRs develop PAN^36^. These observations indicate complex etiological roles of aging, sex, hypertension, and genetic backgrounds in the development of PAN in laboratory rats.

Our BNP knockout rat lines are derived from the Dss strain and are maintained on a low salt diet^19^. Thus, our BNP-defective rats are predisposed to salt-sensitive hypertension and are PAN-prone through their Dss background. Nevertheless, the diet-independent, hypertension-independent, progressive and early onset arteritis was specific to female BNP knockouts. However, the salt sensitivity of the Dss rat is not unique to a single mutation, raising the possibility of modifiers confounding the phenotype of Nppb−/− females. In light of this limitation, we are currently assessing the influence of genetic dosage and genetic backgrounds on the PAN phenotype in order to address any confounding genetic variation inherent in the Dss Nppb−/−, through establishing Nppb+/+, +/−, −/− rats on non-hypertensive Wistar background.

Mesenteric nodes were characterized by intense arterial wall thickening, medial hypertrophy, and progressive inflammation. Although fibrin-plugs, expanding fibroblasts, infiltrating immune cells, and a wide-spread presence of inflammatory cytokines were noted in the lesions by 9 months, increased arterial wall mass was the major pathological change visible at 4 months, suggesting a late inflammatory response, secondary to the intensive arterial wall thickening. These observations suggest that BNP’s primary target is suppression of medial (and to lesser degree intimal) hyperplasia and hypertrophy, and subsequent hindrance of fibrotic remodeling and inflammation. These effects are reminiscent of BNP’s well-established protective effects against cardiac remodeling, including those against cardiac hypertrophy, fibroblast proliferation, and induction of fibrosis-related gene, Tgfβ^19^,^20^,^21^.

The vasoprotective effects of guanylyl cyclase agonists, especially nitric oxide (NO, soluble GC agonist) and CNP (GC-B agonist), are well established. NO prevents platelet aggregation and adhesion^37^,^38^, smooth muscle cell proliferation and migration via cGMP-dependent and independent mechanisms^39^,^40^,^41^, and extracellular matrix proliferative changes, such as Tgfβ expression^42^,^43^,^44^. Likewise, CNP inhibits intimal thickening in response to smooth muscle cell migration in animal models of atherosclerosis^45^,^46^,^47^. Moreover, disruption of endothelial-specific expression of CNP leads to endothelial dysfunction, atherogenesis, and aneurysms in mice, although these CNP-dependent vasoprotective functions appear to be mediated by the cGMP-independent NPR-C receptor^30^. Of note, BNP binds both GC-A and NPR-C. We postulate that BNP may engender a similar vasoprotective role, likely, to some extent, redundant with those of NO and CNP, through cGMP induction or through NPR-C activation. Be that as it may, BNP knockout rats have both NO and CNP pathways intact. Therefore, it is plausible that BNP plays a more specific, and necessary role in maintaining the integrity of small arteries, such as mesenteric arteries, particularly in females.

One puzzling question remaining is the crucial timing for BNP protection of young females in the rodent. As described above, endogenous female hormones often have cardioprotective effects^48^,^49^. Moreover, various studies have demonstrated the link between hormone replacement therapy (HRT) and reduced intima-media thickness of carotid arteries in aged women^50^,^51^, suggestive of the beneficial effects on arterial remodeling. However, some of the notable adverse effects of HRT include an increased risk of deep vein thrombosis, atherosclerosis, and stroke^52^. We therefore postulated that BNP deletion-mediated arteritis was worsened by enhanced coagulation in females. However, we did not see evidence of enhanced coagulation in the BNP-defective females. As this explanation is purely speculative, a follow-up study of Wistar-Nppb−/− females, as well as pathway analysis of cardiac and mesenteric smooth muscle cells would provide a mechanistic understanding. Further studies in Nppb−/− rats, with or without ovariectomy, with controlled NO/eNOS, CNP and/or NPR-C systems, would help illuminate the vasoprotective effects of BNP, separate from the influence of female hormones and other sources, and better illustrate the mechanism through which the BNP effect is occurring.

From our data, it is plausible that defects in the BNP-GC-A-cGMP axis (or the BNP-NPR-C pathway) may play a role in early onset arteritis in women. In humans, PAN is a systemic necrotizing and inflammatory arterial disease, largely involving vessel branch points. PAN lesions result in micro-aneurysms, thrombi, and tissue ischemia, with hypertension in 25–31% of patients^53^,^54^. However, men are twice as likely to be affected as women; thus, though phenotypically similar in presentation, the Nppb−/− female does not faithfully recapitulate the human disease condition of PAN. Other arterial diseases are known to be more predominant in women than men, such as systemic lupus erythematosus (SLE). Targeting then, the BNP-GC-A-cGMP axis may offer an alternative therapeutic option for vascular diseases, in particular, females. A recent study detailing the murine model of lupus, the MRL/lpr strain, linked the clinical hallmarks of SLE; progressive inflammation, organ damage and dysfunction, to excessive cGMP catabolism^55^. Pharmacological inhibition of targeted PDE1 and restoration of cGMP levels significantly lowered peripheral hypercellularity, blunting disease progression. This example illustrates an arteriopathy, where therapy was linked to restoration of the GC-A/cGMP pathway. We suggest then that the BNP/GC-A/cGMP/PDE system may represent a modifier in arterial disease onset or severity as is the case in the MRL/lpr mouse, and screening of clinical populations for expression differences, or genetic disruption of the BNP/GC-A/cGMP/PDE system could aid in developing a mechanistic understanding for many idiopathic rare arteriopathies.

Segmental arterial mediolysis (SAM) shows similar pathological characteristics as PAN. However, SAM typically presents intra-abdominal hemorrhage in aged patients, with lysis of medial layer of arterial wall resulting in fusiform aneurysms, stenosis and occlusions within splanchnic, and some cases in renal, arterial branches. In SAM, arterial lesions are not associated with inflammation. In contrast, female Nppb−/− rats did not show abdominal hemorrhage and aneurysms and occlusions within renal arterial branches, while demonstrating fusiform aneurysms and occlusions within mesenteric and, to a lesser extent, splanchnic arteries^56^,^57^. The mesenteric lesions often involved progressive inflammation. Accordingly, we concluded that the vasculitis found in our Nppb−/− rats as PAN-like, rather than SAM.

In conclusion, our data suggests a sexual dimorphic, vasoprotective role for BNP. Thus, defects in the BNP/GC-A/cGMP pathway may play a role in arteriopathies in women, while GC-A agonists may provide effective therapy for such conditions. Additionally, our BNP defective rat would serve as a useful small animal model to evaluate therapeutic options for small artery remodeling.

## Methods

All animal procedures were approved by the Mayo Clinic Institutional animal care and use committee and were carried out in accordance with the approved guidelines.

### Animals

Nppb−/− rats were generated on Dahl Salt Sensitive (Dss) backgrounds as described previously, under the official strain symbol of SS-Nppb^em2Mcwi^ (m2), and SS-Nppb^em4Mcwi^ (m4)^19^,^25^. Briefly; Nppb−/− M2 and M4 lines have deletions of 100 bp and 138 bp bracketing intron 1 and exon 2 as previously detailed^19^,^25^. A total of 28 Nppb−/− (of both M2 and M4 genotypes), broken down into the following; 14 Nppb−/− followed for 9 months, 10 Nppb−/− followed for 3 months, and an additional 4 Nppb−/− for the 4 month time point. A total of 12 Nppb+/+ female rats were used, 6 rats for the survival study, and an additional 6 Nppb+/+ rats for the 3 month time point

### Tissue harvesting, RNA extraction

At study termination, rats were anesthetized with isoflurane, jugular vein bled, and then euthanized with carbon dioxide. Organs were removed, and either frozen in 2 mg pieces in 1.5 mL SC Micro Tube, Sarstedt (Numbrecht, Germany) and stored at −80 °C for later mRNA extraction, or frozen in Tissue-Tec OCT Compound (Miles, Elkhart, IN) freezing media, and stored at −80 °C.

For histology, organs were brought to −20 °C overnight, and cross-sectioned in a cryostat at −20 °C, in 7–12 μm thick sections, then subjected to hematoxylin and eosin staining (American Master Tech, Lodi, CA) as per manufacturer’s instructions. Masson Trichrome Staining was performed as per manufacturer’s instructions with collagen content quantified as previously reported^20^. Sections were imaged by a light microscope (Axioplan). Histopathology was determined by veterinary consult, blinded to experimental groups.

For RNA extraction, 1 mg of frozen tissue was homogenized in TRIzol (500 ul; GIBCO BRL, Gaithersburg, MD) and then subsequent phenol-chloroform extraction according to manufacturer’s instructions. Two μg of total RNA was then reverse transcribed into cDNA by RNA to cDNA EchoDry Premix (Clontech Laboratories, Mountain View, CA) and the resulting cDNA was mixed with gene-specific primers and assayed by quantitative-PCR (qPCR).

For qPCR; 1 μl of cDNA were added to 2.5 μl of a 10× Primer assay (Supplemental Table 1) and 12.5 μl of 2× Master Mix FastSYBR® Green (Applied Biosystems, CITY and STATES), and 9 μl of sterile water was added to reach a final volume of 25 μl. Amplification reactions were performed in a 7300 Real Time PCR System (Applied Biosystems) following the conditions of standard protocol for FastSYBR Green® (same for all genes). After denaturation of the cDNA and enzyme activation at 95 °C for 5 minutes, 40 cycles were performed in 2 steps (95 °C for 10 seconds and 60 °C for 30 seconds). The expression of Glyceraldehyde 3-phosphate dehydrogenase (GAPDH) and Beta Actin were employed as housekeeping genes where appropriate. Expression levels are presented as relative transcripts to the appropriate house keeping genes’ expression levels as described in the figure legends.

### Hematology and urinalysis

Rats were placed in metabolic cages with free access to tap water and food pellets. Overnight urine volume, creatinine and protein excretion were measured. For hematological parameters animals were bled as previously described^19^, serum separated, and assayed for Thrombin-Anti Thrombin Complex, Activated Partial Thromboplastin Time, and Citrate Prothrombin Time as per manufacturer’s protocol (IDEXX Coag Dx, IDEXX laboratories), on a Coag Dx Analyzer (IDEXX Laboratories).

### Non-invasive BP measurement

Blood pressure is measured by tail-cuff using the CODA high throughput non-invasive BP system (Kent Scientific), detailed previously^21^,^58^. Briefly, the animal is placed in individual holders at least 10 minutes prior to obtaining pressure measurements. Acclimatization is accomplished through training sessions. BP measurements were taken every two weeks throughout the duration of the survival study (9 months). A minimum of 10 readings were taken, and the mean of acceptable readings (as determined by the software instrument) were recorded per animal, at each observation time.

Echocardiography (ECHO) for non-invasive assessment of cardiac function and structure. Standard ECHO was performed on surviving genotypes at 9 months. All ECHO examinations and analysis were performed by a skilled sonographer (S.H) blinded to treatment, as previously described^19^,^21^. Briefly; a Vivid 7 ultrasound machine (General Electric, Waukesha, WI) with a GE Vivid 7 10S transducer was used to collect ECHO data. Images were taken at a depth of 2–2.5 cm of the parasternal short-axis left ventricular view, using the papillary muscles as anatomical landmarks. Images were then exported to a separate workstation for analysis using EchoPAC (General Electric, Waukesha WI).

### Confocal microscopy

Sections were analyzed by immunofluorescence using antibodies against desmin (Anti-desmin Antibody, clone DE-B-5, Millipore), basic fibroblast growth factor 2 (FGF-2 antibody (G-2) sc-365106, Santa Cruz Biotechnology), alpha smooth muscle actin (anti- α-SMA, ab5694, abcam), podocin (rabbit anti-podocin antibody, Sigma), IL6 (anti-IL6, ab6672, abcam), IL8 (anti-IL8 antibody, 710256, Invitrogen), CD45 (anti-CD45 (H30), sc-25590, Santa Cruz Biotechnology), and DAPI, nuclear probe 4′, 6-diamidino-2-phenylindole-2HCl (Invitrogen).

Fluorescent images were obtained with a Laser Scanning Microscope 510 (Zeiss, Thornwood, NY) and analyzed using Zeiss Laser Scanning Microscope Image Browser. OCT-embedded mesenteric and renal sections were cryosectioned, mounted on frosted slides, and air dried. Before the incubation with primary antibodies, sections were fixed in acetone at −20 °C for 20 minutes, air dried, and washed in Phosphate Buffered Saline (PBS). To prevent nonspecific binding of immunoglobulins (IgGs), tissues were blocked for 1 h (PBS/1% fetal bovine serum). Sections were incubated with primary antibodies diluted 1:300 in blocking solution overnight at 4 °C. Day 2, samples were washed 3 times (10 minutes each) with PBS and incubated with secondary antibodies at 1:1000 in blocking solution (Alexa Fluor 568 donkey anti-mouse, Alexa Fluor 594 donkey anti-goat, Alexa Fluor 647 donkey anti-rabbit IgG) for 1 h at room temperature. Next, tissues were washed 3 times (5 minutes each) with PBS, and then incubated with DAPI (1:3000) for 5 minutes at room temperature. Afterwards, slides were washed with PBS and then mounted with Dako Fluro Mounting Media (Dako, Carpinteria, CA).

### Statistical analysis

Comparisons between groups were made with unpaired Student’s t-tests and f-tests where appropriate. Comparisons of BP values between groups were performed by two-way ANOVA, and Sidak’s multiple comparison test for repeated measurements. Survival curves were compared by log-rank (Mantel-Cox) test. Data are expressed as mean ± standard error. Significance was predetermined as p < 0.05 and n refers to sample size.

## Additional Information

**How to cite this article**: Holditch, S. J. *et al*. Arterial Remodeling in B-Type Natriuretic Peptide Knock-Out Females. *Sci. Rep*. **6**, 25623; doi: 10.1038/srep25623 (2016).

## Supplementary Material

Supplementary Information
