# Supplementary material for: Arterial Remodeling in B-Type Natriuretic Peptide Knock-Out Females
Source: Sci Rep. 2016 May 10;6:25623. doi: 10.1038/srep25623 (PMC4861904; doi:10.1038/srep25623)
Supplement: Supplementary Information [file srep25623-s1.pdf]

Supplementary Information

"ARTERIAL REMODELING IN B-TYPE NATRIURETIC PEPTIDE KNOCK-OUT FEMALES"

Short title: Vasoprotective role of BNP

Sara J. Holditch[1] Ph.D, Claire A. Schreiber[1] B.S, John C. Burnett[2] M.D, Yasuhiro Ikeda[1] Ph.D, D.V.M

Supplementary Table S1. Quantitative Real Time PCR Primer List

Gene Name	Gene Abbreviation	Species	Cat No.	Primer Assay
Atrial Natriuretic Peptide	Nppa	Rat	QT00366170	Rn_RGD:3193_1_SG QuantiTect Primer Assay
B-Type Natriuretic Peptide	Nppb	Rat	QT00183225	Rn_Nppb_1_SG QuantiTect Primer Assay
Natriuretic peptide receptor 1	Npr1	Rat	QT00178339	Rn_Npr1_1_SG QuantiTect Primer Assay
Natriuretic peptide receptor 2	Npr2	Rat	QT00187432	Rn_Npr2_1_SG QuantiTect Primer Assay
Collagen type 1 alpha 1	Col1a1	Rat	QT01081059	Rn_Col1a1_1_SG QuantiTect Primer Assay
Transforming growth factor beta	Tgfb	Rat	QT00187796	Rn_Tgfb1_1_SG QuantiTect Primer Assay
Fibronectin 1	Fn1	Rat	QT00179333	Rn_Fn1_1_SG QuantiTect Primer Assay
Tissue inhibitor metalloprotease 1	Timp1	Rat	QT00185304	Rn_Timp1_1_SG QuantiTect Primer Assay
Glyceraldehyde 3-phosphate dehydrogenase	GAPDH	Rat	QT00199633	Rn_Gapd_1_SG QuantiTect Primer Assay
Alpha cardiac actin 1	Actc1	Rat	QT01081185	Rn_Actc1_1_SG QuantiTect Primer Assay
Tropomyosin 1	Tpm1	Rat	QT00194264	Rn_Tpm1_1_SG QuantiTect Primer Assay
Transthyretin	Ttr1	Rat	QT01081304	Rn_Ttr_1_SG QuantiTect Primer Assay
Myosin heavy chain 7	Myh7	Rat	QT00189504	Rn_Myh7_1_SG QuantiTect Primer Assay
Beta Actin	Actb	Rat	QT00193473	Rn_Actb_1_SG QuantiTect Primer Assay
Vascular endothelial growth factor	VegF	Rat	QT00198954	Rn_RGD:619991_1_SG QuantiTect Primer Assay
Jagged 1	Jag1	Rat	QT00193424	Rn_Jag1_1_SG QuantiTect Primer Assay
Apolipoprotein E1	Apoe1	Rat	QT00408373	Rn_Apoe_1_SG QuantiTect Primer Assay
Nephrosis 1	Nphs1	Rat	QT00189805	Rn_Nphs1_1_SG QuantiTect Primer Assay
